# Are gravitational wave ringdown echoes always equal-interval?

**DOI:** 10.1140/epjc/s10052-018-5974-y

**Published:** 2018-06-11

**Authors:** Yu-Tong Wang, Zhi-Peng Li, Jun Zhang, Shuang-Yong Zhou, Yun-Song Piao

**Affiliations:** 1 School of Physics, University of Chinese Academy of Sciences, Beijing, 100049 China; 2 Department of Physics and Astronomy, York University, Toronto, ON M3J 1P3 Canada; 3 Interdisciplinary Center for Theoretical Study, University of Science and Technology of China, Hefei, 230026 Anhui China; 4 Institute of Theoretical Physics, Chinese Academy of Sciences, P.O. Box 2735, Beijing, 100190 China

## Abstract

Gravitational wave (GW) ringdown waveforms may contain “echoes” that encode new physics in the strong gravity regime. It is commonly assumed that the new physics gives rise to the GW echoes whose intervals are constant. We point out that this assumption is not always applicable. In particular, if the post-merger object is initially a wormhole, which slowly pinches off and eventually collapses into a black hole, the late-time ringdown waveform exhibit a series of echoes whose intervals are increasing with time. We also assess how this affects the ability of Advanced LIGO/Virgo to detect these new signals.

## Introduction

Recently, the LIGO Scientific and Virgo Collaborations, using ground based laser interferometers, have detected gravitational wave (GW) signals of binary black hole (BH) [1] and binary neutron stars [2] coalescences, which opened a new window to probe gravity physics, particularly in the strong field regime, and the origin of universe.

Inflation is the current paradigm of the early universe. The domain-wall bubbles (or relevant objects) can spontaneously nucleate in de Sitter space and be stretched by the inflation to astrophysical scales [3], see also [4]. In Refs. [5, 6], it has been argued that under certain conditions the interior of a large bubble will develop into a baby universe, which is connected to the exterior region through a wormhole (WH), see also [7]. The throat of the WH is dynamic, which will pinch off shortly after the WH enters into the cosmological horizon, or see [8]. The resulting BHs might be candidates for seeding the supermassive objects at the center of galaxies [9]. Thus, it is possible that the primordial WHs, created and enlarged in the inflationary phase, might be slowly pinching till today, and merge with another compact object (a neutron star or BH). In any case, one could speculate a scenario where a WH may appear as an intermediate state in the coalescences of some compact objects (BH/BH, WH/BH, WH/WH mergers, etc.).

In this paper, we will show that if the post-merger object is a WH, which is slowly pinching off (and eventually will collapse into a black hole), the late-time ringdown waveform will exhibit a series of interval-increasing echoes. It is commonly assumed, after Cardoso et.al.’s seminal work Refs. [10, 11], that the intervals between the neighboring GW echoes are constant, which has been widely used in searching for the signals of echoes in GW data [12–14]. However, this assumption could bring bias that causes systematic errors in the parameter estimation of signals, e.g. [15], as we find that the GW echoes may not be equal-interval. Our result suggests a more general pool of templates for the echo searches might be desirable.

## Setup and ringdown echoes

**Fig. 1:**
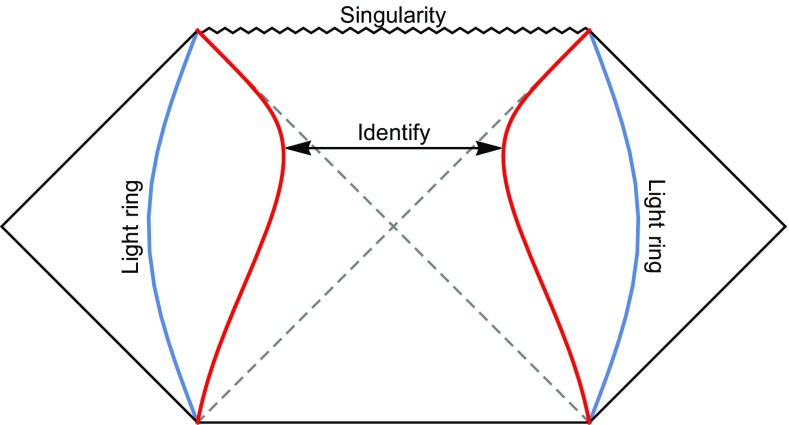
The conformal diagram of a slowly pinching WH. The WH is constructed by gluing two Schwarzschild space-time at $r=r_0(t)$ (the red lines), which start at somewhere inside the light ring and end at the Schwarzschild radius at a finite *t*. The blue lines show the light rings of the Schwarzschild metrics

Let us begin with the spacetime depicted by Fig. 1, where the post-merger object is initially a WH, which slowly pinches for a period before collapsing into a BH. Here, for our purposes, we make use of the simple phenomenological model, the Morris–Thorne WH [16, 17], which is obtained by gluing the Schwarzschild metrics

1 $$\begin{aligned} ds^{2}=-B dt^{2}+{dr^{2}\over B}+r^{2}d\Omega ^{2},\quad \left(B=1-{2M\over r}\right) \end{aligned}$$

of both sides at $r=r_{0}>2M$, where $r_{0}$ is the radius of the throat, see e.g. [18] for the stability of the Morris–Thorne WH.

We work with the tortoise coordinate $|dr/dr_{*}|=B$. Generally, we define $r_*(r_0)=0$, and will have $r_*>0$ and $r_*<0$ for both sides at the throat, respectively. To illustrate the GW waveforms, we scatter a test wave packet, which satisfies the Klein–Gordon equation in the pinching WH background $\Box \Phi =0$. We expand $\Phi$ as $\Phi =\Sigma _{lm}{Y_{lm}(\theta ,\phi )\over r}\Psi _{lm}(r)$, and get the Regge–Wheeler equation

2 $$\begin{aligned} \left[-\frac{\partial ^{2}}{\partial t^{2}}+\frac{\partial ^{2}}{\partial r_{*}^{2}}-V_{l}(t,r_*)\right]\Psi _{lm}(t,r_*)=0 , \end{aligned}$$

with

3 $$\begin{aligned} V_l(t,r_*)= \left\{ \begin{array}{ll} V_l^{BH}(r_*-L/2) &{}\quad \text {for} \quad r_*>0, \\ V_l^{BH}(-r_*-L/2) &{}\quad \text {for} \quad r_*<0, \end{array} \right. \end{aligned}$$

where $V_l^{BH}(r_*(r))$ is the barrier $V_l^{BH}(r)=B \left[\frac{l(l+1)}{r^{2}}+\frac{B'}{r}\right]$ of BH but written in the coordinate $r_*^{BH}$. As an illustration, we will focus on $l= 2$ in the following. In Schwarzschild-like WH background, what $\Phi$ feels is a pair of mirror potentials $V_l^{BH}(r_*(r))$ glued at $r_0$ ($r_*=0$), and the separation between the barriers of mirror potentials is

4 $$\begin{aligned}&L\simeq 2\int _{r_{0}}^{3M}\frac{dr}{B}\simeq 4M\log \left[\frac{M}{\ell (t)}\right], \nonumber \\&\quad \, \mathrm{for}\,\, \ell (t)=r_{0}-2M\ll M, \end{aligned}$$

which will slowly get longer for ${\dot{\ell }(t)}< 0$. When $\ell (t)=0$, $r_0$ equals to the Schwarzschild radius, and the WH becomes a BH.

**Fig. 2:**
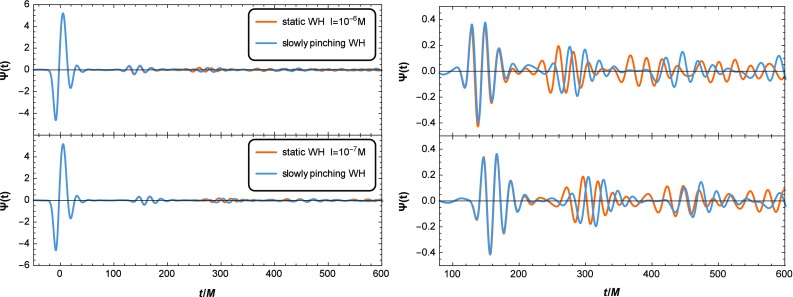
Ringdown waveforms of post-merger objects, which correspond to the static and slowly pinching WHs (depicted by Fig. 1), respectively. The right panel is equivalent to the left panel in the segment $100\leqslant t/M\leqslant 600$

It has been found in Refs. [10, 11] that if the post-merger object is a WH, the ringdown waveform will consist of the primary signal (almost identical to that of BH) and a series of equal-interval echoes, see also [19, 20]. Considering the pinching of WH is enough slow, we solve Eq. (2) with the initial Gaussian perturbation

5 $$\begin{aligned} \frac{\partial \Psi _{lm}}{\partial t}(0,r)=e^{-(r_{*}-r_{g})^{2}/\sigma ^{2}},\quad \, \Psi _{lm}(0,r)=0, \end{aligned}$$

where $r_{g}=10M$, $\sigma =6M$. We plot the corresponding waveforms in Fig. 2, and see that contrary to Refs. [10, 11], the interval $\Delta t_{echo}$ of the echoes in our scenario are not equal, but increase with time. The shift of interval following the *i*th echo $\delta t_i$ is approximately

6 $$\begin{aligned} \delta t_{i}=\Delta t^{i+1,i}_{echo}-\Delta t^{i,i-1}_{echo}\sim 8M\log \left[{\ell (t_i)\over \ell (t_{i+1})}\right], \end{aligned}$$

where $\Delta t^{i+1,i}_{echo}$ is the interval $\Delta t_{echo}$ between the $(i+1)$th and *i*th echoes.

We will estimate the quasinormal frequencies (QNFs) in slowly pinching WH background. We focus on a period $t\simeq 2L$, during which the separation *L* between the barriers will become ${\tilde{L}} =L+\Delta L$. In the approximation $\Delta L\ll L$, we could regard the moving of barriers as the perturbation for the QNFs $\omega _{L}$, which will give rise to the shifts of $\omega _{L}$ to $\omega _{{\tilde{L}}}$. Thus in the frequency domain, we can write Eq. (2) as

7 $$\begin{aligned} \left[\frac{\partial ^{2}}{\partial r_{*}^{2}}+{\omega }^2_{L}-V_{l}(L,r_*)\right]{\hat{\Psi }}_{lm}=0, \end{aligned}$$

where

8 $$\begin{aligned} {\omega }^2_{L}=\omega ^2_{\tilde{L}}-{\partial \omega ^2_L\over \partial L}\Delta L, \end{aligned}$$

and $\Psi (t,r_*)=\int \frac{d\omega }{2\pi } {\hat{\Psi }}(\omega ,r_*)e^{i\omega t}$. In Eq. (7), the separation between the barriers is still *L*, the effect of $\Delta L$ ($\ll L$) is absorbed into $\omega _{L}^2$. Equation (7) is the Regge–Wheeler equation for the static WH, and its QNFs have been calculated in Ref. [21],

9 $$\begin{aligned} \omega _{L,n}= {n\pi \over L}+{i\ln \left|R_{BH}(\omega _{L,n})\right|\over L}, \end{aligned}$$

where $R_{BH}(\omega _{L,n})$ is the reflection coefficient of the barrier $V_l^{BH}(r_*)$. Considering the expansion

10 $$\begin{aligned} R_{BH}(\omega _{L,n})=-1 +\sum _{j=1}^{\infty } {R^{(j)}_{BH}(0)\over j!} \omega _n^k, \end{aligned}$$

we have

11 $$\begin{aligned} {Re}({\omega }_{L,n})\simeq { n\pi \over L},\quad { Im}({\omega }_{L,n})={\ln \left|R_{BH}(\omega _{L,n})\right|\over L}\lesssim \mathcal{O}({1\over L^3}). \end{aligned}$$

According to Eq. (8), we have

12 $$\begin{aligned} \omega ^2_{{\tilde{L}},n}\simeq \omega _{L,n}^2-2\omega ^2_{{L},n}{\Delta L\over L}, \end{aligned}$$

where $\omega _{{L},n}\simeq n\pi /L$ is used.

Generally, after the primary signal is reflected off the barrier on the other side, the corresponding signal will consist of a sum of WH QNMs, e.g. [21]. We find that in slowly pinching WH background, after a period $t\simeq 2{L}$, the QNFs reduce to

13 $$\begin{aligned} {Re}({\omega }_{{\tilde{L}},n})\simeq { n\pi \over L}\left(1-{\Delta L\over L}\right). \end{aligned}$$

Thus

14 $$\begin{aligned} \Psi (t)\sim & {} \sum _{n=-\infty }^{\infty }c_{n}e^{-i({{ n}\pi \over L}-{{n}\pi \Delta L\over L^2})t} e^{\mathrm{Im}(\omega _{L, n})t}\nonumber \\= & {} \sum _{n=-\infty }^{\infty }c_{ n}e^{-i2n\pi ({t\over \Delta t_{echo}})} e^{\mathrm{Im}(\omega _{L, n})t} \end{aligned}$$

with the period

15 $$\begin{aligned} \Delta t_{echo}\simeq 2L/\left(1-{\Delta L\over L}\right). \end{aligned}$$

Thus the signal will be repeated periodically ( referred to as “echoes” in the literature). However, since the WH is slowly pinching off, we actually have $L_{i+1}=L_{i}+\Delta L_{i}$ in successive period $t\simeq 2L_i$, so

16 $$\begin{aligned} \Delta t^{i+1,i}_{echo}\simeq \Delta t^{i,i-1}_{echo}/\left(1-{\Delta L_{i}\over L_i}\right)>{\Delta t^{i,i-1}_{echo}}. \end{aligned}$$

Replacing $\Delta t_{echo}$ in (14) with $\Delta t^{i+1,i}_{echo}$, we will obtain a waveform $\Psi (t)$ with the interval-increasing echoes. Considering $\delta t_{i}=\Delta t^{i+1,i}_{echo}- \Delta t^{i,i-1}_{echo}$ and $2L_i=\Delta t^{i,i-1}_{echo}$, we have $\delta t_{i}\simeq 2\Delta L_{i}$, which is consistent with Eqs. (4) and (6).

## Effect of the interval shift

We will assess and discuss the effect of the shift of echo interval on the search for the signals of echoes in GW data. Based on Eqs. (14) and (16), the GWs ringdown waveform in Fig. 2 is modelled as

17 $$\begin{aligned} \Psi (t)= & {} \Psi ^{BH}(t)+\Psi ^{echo}(t)\nonumber \\= & {} {\mathcal{A}}e^{- t/{\tau }}\cos (2\pi {f}t+{\phi })\nonumber \\&+\,\sum _{n=1}^{{\tilde{N}}_{echo}}(-1)^{n}\mathcal{A}_{n}e^{-\frac{x^2_{n}}{2 \sigma ^2_{n}}}\cos (2\pi {f}_n x_n), \end{aligned}$$

where $\Psi ^{BH}(t)$ is the post-merger BH-like signal with the amplitude $\mathcal{A}$ and the damping time $\tau$, and $\Psi ^{echo}(t)$ is the echo signal with the amplitude $\mathcal{A}_n\sim \frac{1}{3+n} {\mathcal{A}}$, which is modulated by a Gaussian profile with the width $\sigma _n$, and $x_n = t-\sum _{i=0}^n\Delta t^{i+1,i}_{echo}$.

**Fig. 3:**
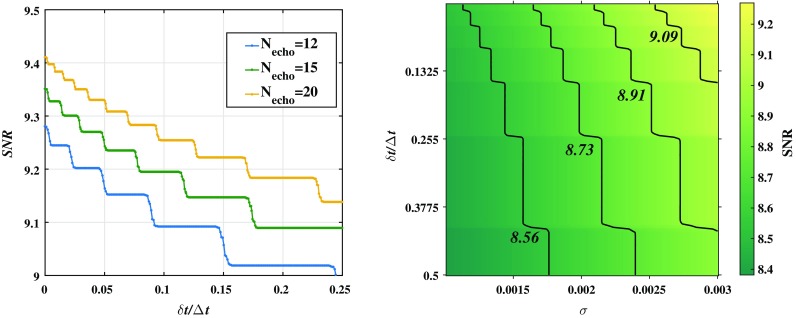
Left panel: the SNR with respect to $\delta t/\Delta t$ for different $N_{echo}$. Right panel: the SNR with respect to $\sigma$ and $\delta t/\Delta t$, we fix $N_{echo}=12$

When the signal and (17) are maximally matched, the expected matched-filter SNR is [22, 23]

18 $$\begin{aligned} \rho = \sqrt{4\int _{0}^{\infty }df\frac{|{\tilde{\Psi }}(f)|^2}{S_{n}(f)}}, \end{aligned}$$

where ${\tilde{\Psi }}(f)=\int \Psi (t)e^{-2\pi i ft}dt$, and $S_{n}(f)$ is the noise power spectral density (PSD) of detector. We focus on the GW1509014 event ($M\simeq 68M_{\bigodot }$), which yields ${f}\simeq 250$Hz and ${\tau }\simeq 4\times 10^{-3}$s in (17) [24]. We also choose $\mathcal{A}\simeq 6\times 10^{22}$, which is consistent with the best-fit parameters for the GW150914 event. We, for simplicity, set all $\sigma _n$ ($=\sigma$) as well as $\delta t_i$ ($=\delta t$) equal, and have

19 $$\begin{aligned} x_n=t-(n+1)\Delta t_{echo}^{1,0}-\frac{n(n+1)}{2}\delta t. \end{aligned}$$

Regarding the post-merger object of GW150914 as a pinching WH, we have $\Delta t^{1,0}_{echo} \simeq 3\times 10^{-2}$s for initial $\ell (t)\sim 10^{-5}$.

We calculate the SNR in a fixed segment $T=N_{echo}\Delta t\sim 3N_{echo}\times 10^{-2}$s. We plot the SNR with respect to $\delta t/\Delta t$ in left panel of Fig. 3, where $\Delta t=\Delta t_{echo}^{1,0}$ is set. We take $N_{echo}=20$ (so $T\simeq 3N_{echo}\times 10^{-2}$s$=0.6$s), and see that if all echoes are equal-interval ($\delta t=0$), i.e.${\tilde{N}}_{echo}=N_{echo}$ for (17), we have the SNR $\rho \simeq 9.4$, but if $\delta t/\Delta t\simeq 0.1$, the SNR will reduce to $\rho \simeq 9.3$, since we only have ${\tilde{N}}_{echo}\simeq 12$ in this segment. Thus the larger is $\delta t/\Delta t$, the less is the number of echoes in fixed segment, so the lower is the SNR. In right panel of Fig. 3, we also show how the different values of $\delta t/\Delta t$ alter the SNR of the signals with the echo width $\sigma$.

The shift of echo interval is encoded in $\delta t$. The LIGO/Virgo collaborations modelled the ringdown waveform without the echoes as $\Psi ^{BH}(t)$, see (17), and found the SNR $\rho \sim 8$ [24]. Generally, the inclusion of echoes will enhance the SNR, e.g. [15]. Our result indicates that the shift of echo interval could significantly affect the parameter estimation of echo signals, when one searched for the corresponding signals in GW data.

## Discussion

Even though after the merger a BH/BH binary (or BH/WH binary) eventually develop into a BH, an exotic intermediate state might exist. We show that if such a state is a WH, which is slowly pinching off (and eventually will collapse into a BH), the ringdown waveform will exhibit a series of echoes, as pointed out in [10]. However, we have found that the usual assumption that the GW echoes are equal-interval is not always applicable. In particular, in our scenario the intervals between the neighboring echoes will increase with time. We have argued the significant effect of the shift of echo interval on the search for the signals of echoes in GW data released by LIGO/Virgo.

The viability of WH depends on special models, which is still a developing subject, e.g. [25–28]. Some of the issues might be better understood by performing numerical simulations of binary mergers with WHs. The physics of GW echoes has recently been extensively studied, see also [29–31]. While the post-merger object we considered is a WH, our result may also be applicable for other exotic compact objects (e.g. [32–34]), as well as the BHs with the correction of modified/quantum gravity [35, 36], with the shift of their reflector surface towards the Schwarzschild radius. However, if initial state is not a BH, the inspiral stage could in principle be used to discriminate against a two-BH initial state, since the quadrupole moment, tidal love numbers or absorption of the initial state is different from that of a BH, see e.g. [37, 38].
